# Organoids as complex (bio)systems

**DOI:** 10.3389/fcell.2023.1268540

**Published:** 2023-08-25

**Authors:** Tiago G. Fernandes

**Affiliations:** ^1^ Department of Bioengineering and iBB—Institute for Bioengineering and Biosciences, Instituto Superior Técnico, Universidade de Lisboa, Lisbon, Portugal; ^2^ Associate Laboratory i4HB—Institute for Health and Bioeconomy, Instituto Superior Técnico, Universidade de Lisboa, Lisbon, Portugal

**Keywords:** complex systems, organoids, stem cells, systems biology, disease modeling

## Abstract

Organoids are three-dimensional structures derived from stem cells that mimic the organization and function of specific organs, making them valuable tools for studying complex systems in biology. This paper explores the application of complex systems theory to understand and characterize organoids as exemplars of intricate biological systems. By identifying and analyzing common design principles observed across diverse natural, technological, and social complex systems, we can gain insights into the underlying mechanisms governing organoid behavior and function. This review outlines general design principles found in complex systems and demonstrates how these principles manifest within organoids. By acknowledging organoids as representations of complex systems, we can illuminate our understanding of their normal physiological behavior and gain valuable insights into the alterations that can lead to disease. Therefore, incorporating complex systems theory into the study of organoids may foster novel perspectives in biology and pave the way for new avenues of research and therapeutic interventions to improve human health and wellbeing.

## 1 An overview of complex systems theory

Complex systems can be found in the natural world and in many different man-made inventions, including finance, economics, and social organizations (Waldrop, 1993). The study of such systems is interdisciplinary and focuses on the examination of non-intuitive, adaptive, and dynamic properties. The roots of complex systems theory can be traced back to various scientific fields, including mathematics, physics, biology, ecology, economics, and social sciences. One of the earliest pioneers of complex systems theory was the mathematician Norbert Wiener, who developed the field of cybernetics in the 1940s and 1950s, seeking to understand the feedback mechanisms that govern the behavior of systems (Wiener, 1948; Shannon et al., 1950). Another influential figure was the physicist Murray Gell-Mann, who proposed the concept of “complex adaptive systems” in the 1960s to describe systems that exhibit emergent behavior (Holland, 1992; Gell-Mann, 1995). In the 1970s, the Santa Fe Institute was established as a center for the study of complex systems, and many of the key figures in the field today, including Stuart Kauffman, John Holland, and Brian Arthur, were associated with the institute. Kauffman, in particular, made significant contributions to the field with his work on complex systems in biology (Glass and Kauffman, 1972; Kauffman, 1984). The 1980s and 1990s saw the development of new mathematical and computational tools for the study of complex systems, including chaos theory, fractals, and cellular automata (Mandelbrot and Aizenman, 1979; Pippard, 1982; Wolfram, 1984; Langton, 1986). These tools allowed researchers to simulate and model complex systems and study the emergence of patterns and behaviors (Axelrod and Hamilton, 1981; Forrest, 1990).

**FIGURE 1:**
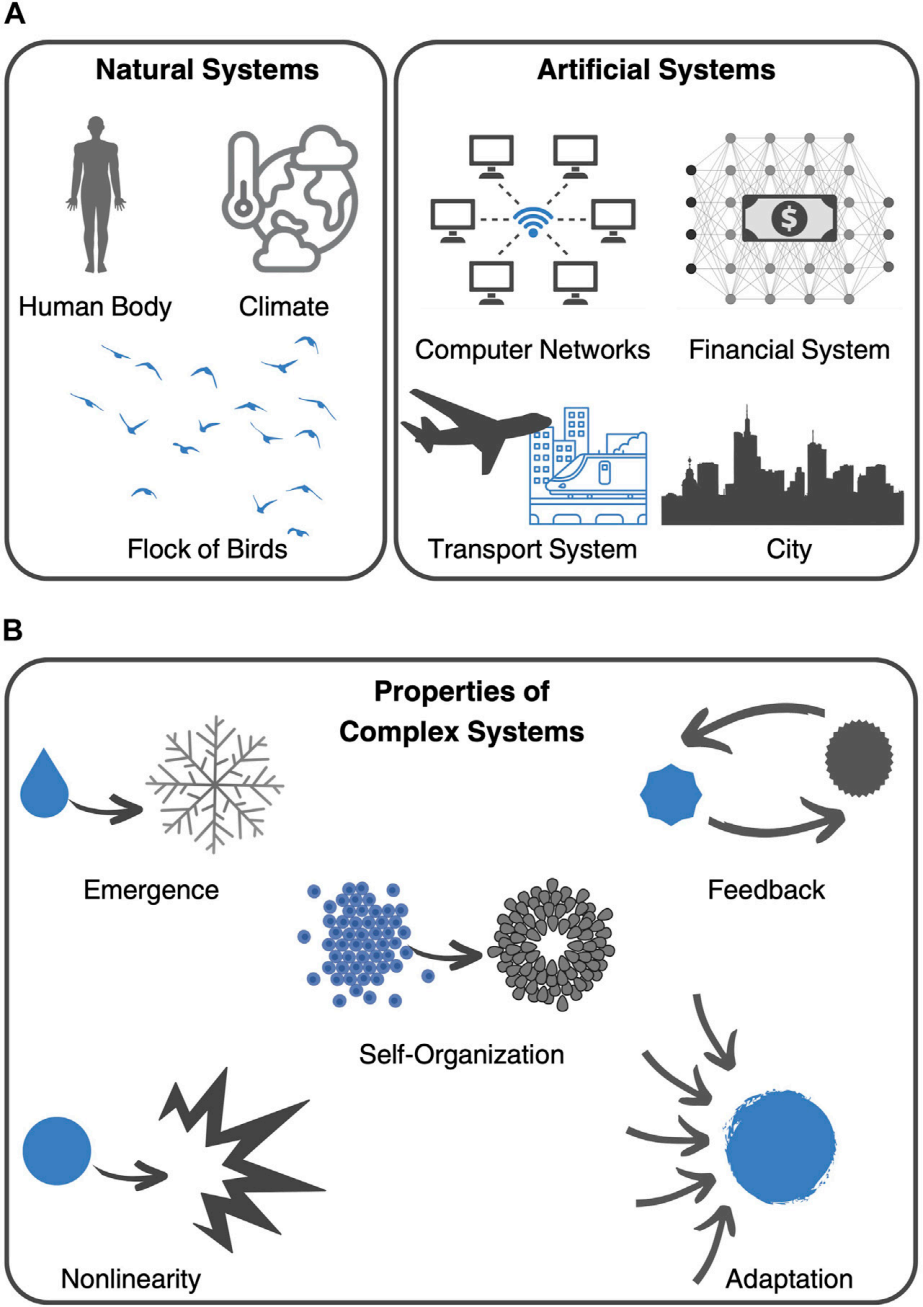
Complex systems. **(A)** Examples of complex systems include natural systems, such as the human body, the climate system, and the collective behavior of flocks of birds, as well as man-made inventions, like computer networks, the financial system, transport systems, and cities. **(B)** Key characteristics, or principles, that define complex systems include emergence, self-organization, feedback, nonlinearity, and adaptation.

Today, complex systems theory continues to be a thriving field of research with applications in a wide range of disciplines. Some of the key challenges facing the field include developing new mathematical and computational models that can capture the complexity of real-world systems and understanding the relationship between individual behavior and system-level outcomes (Siegenfeld and Bar-Yam, 2020). Almost 70 years of research have culminated in the Nobel Prize in Physics, 2021, which was awarded “for groundbreaking contributions to our understanding of complex physical systems” with one-half jointly to Syukuro Manabe and Klaus Hasselmann “for the physical modelling of Earth’s climate, quantifying variability and reliably predicting global warming” and the other half to Giorgio Parisi “for the discovery of the interplay of disorder and fluctuations in physical systems from atomic to planetary scales” (Press release. NobelPrize.org., 2021). Parisi, in particular, became known for his work on the collective behavior of animals such as flocks of birds (Ballerini et al., 2008). Focusing on the collective behavior of starlings, he explored the idea that the movements of individual animals in a group can be influenced by the behavior of neighboring animals. As can be seen by this example, complex systems theory has many different applications in the study of natural systems, as well as in the design of artificial systems, like computer networks and transportation structures (Figure 1A).

In fact, the aforementioned examples share key characteristics, or principles, that make them complex systems. These include emergence, nonlinearity, self-organization, feedback, and adaptation (Figure 1B) (Ma’ayan, 2017). These principles help to explain why complex systems can exhibit such diverse and often unpredictable behaviors. One of such principles is emergence, which refers to the phenomenon where complex behavior arises from interactions among simple components. Emergent properties are often difficult to predict or explain based solely on the properties of the individual components. Furthermore, feedback loops, which are cycles of interaction between components that can lead to self-organization and adaptation, create non-linear dynamics, which means that small changes in one part of the system can have large and unpredictable effects on the system as a whole. Depending on the context, feedback can be positive (amplifying) or negative (dampening). From this complex framework, typically emerges self-organization and adaptation. Self-organization refers to the ability of complex systems to spontaneously form structures or patterns without external direction or control. This phenomenon arises from the interactions between components of the system and can lead to new properties or behaviors. On the other hand, adaptation refers to the ability of complex systems to adjust and evolve in response to changing environments or conditions. While facing external disturbances, adaptive systems can exhibit resilience and robustness.

By understanding the behavior of complex systems, researchers can gain insights into the underlying mechanisms that drive their dynamics, and develop strategies for controlling or optimizing their behavior (Cohen et al., 2022; San Miguel, 2023). In this review, I argue that three-dimensional (3D) organoids derived from stem cells develop the characteristics that make them complex (bio) systems. These organoids offer opportunities to explore complex genetic conditions, and model human development, organogenesis, and pathology (Quadrato and Arlotta, 2017; Kim et al., 2020). However, several limitations exist due to the variability and lack of consistent anatomical organization attained in different organoid systems. This text presents the fundamental principles of “*in vitro* organogenesis” and explores the biological aspects that can be effectively modeled with current methods. Additionally, the text also discusses potential improvements that could make organoids reliable tools for investigating the emergent properties displayed by complex (bio) systems.

## 2 Characterization of organoids as complex (bio)systems

**FIGURE 2:**
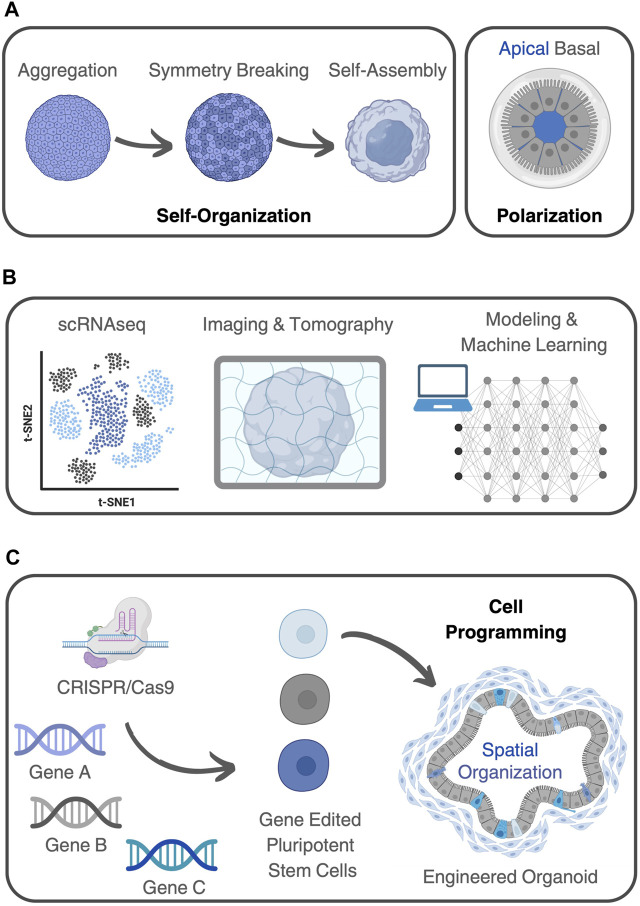
Organoids as complex (bio)systems. **(A)** Organoids demonstrate self-organization, where components within the system arrange themselves into ordered structures. Stem cells can break symmetry and differentiate, giving rise to tissue-specific architecture and functionality. Organoids also show polarization, with cells differentiating along an axis to form structures featuring distinct apical and basal regions. **(B)** Different methods can be used to analyze organoids using a systems-based perspective. These include experimental techniques, like scRNA-seq, various 3D imaging and tomography methods, together with predictive mathematical models, complemented by machine learning algorithms capable of capturing the molecular complexity within living systems. **(C)** Genome editing, using CRISPR-Cas9, for example, can be applied to introduce specific genetic modifications into cells, to investigate the roles of genes, signaling pathways, or cellular behaviors in organoid development and functionality. This capability opens up a range of possibilities for studying organoids and manipulating emergent properties. By selectively editing genes associated with specific pathways or signaling cascades, researchers can program organoid development, behavior, and function.

Organoids, which are three-dimensional *in vitro* cultures that mimic the structure and function of organs (Simian and Bissell, 2017), can indeed be considered complex systems. They meet the criteria of complex systems in a way that they display, if not all, most of the key characteristics or principles that make up such structures (Figure 2A).

Firstly, organoids demonstrate self-organization, where the components within the system arrange themselves into ordered structures without external guidance. In the case of organoids, stem cells can break symmetry and differentiate, giving rise to tissue-specific architecture and functionality (Lou and Leung, 2018). This process is driven by complex signaling pathways and cellular interactions (Yin et al., 2016). Additionally, organoids exhibit emergent behavior, where complex patterns and functionalities arise from the interactions among their constituent cells. Many organoids also show polarization, with cells differentiating along an axis to form structures featuring distinct apical and basal regions. For example, in cerebral organoids, cells self-organize into neural networks displaying cortical layering with distinct neuronal populations arranged in characteristic laminar patterns (Eiraku et al., 2008). These cerebral organoids typically develop and exhibit spontaneous electrical activity, resembling firing patterns seen in the developing brain (Sharf et al., 2022). Likewise, kidney organoids demonstrate differentiation into proximal and distal tubules with specific apical and basal domains (Low et al., 2019). Similarly, intestinal organoids display emergent properties (Tsai et al., 2017), including the formation of crypt-like structures, villi-like protrusions, and the presence of various specialized cell types (Workman et al., 2017).

In fact, embryonic development serves as a classic example of self-organization in biological systems. Cells communicate and coordinate their behaviors to create complex structures and organs. Patterns arise from interactions among cells, including differential gene expression, cell-cell signaling, and mechanical forces (Li et al., 2014). This communication occurs through signaling molecules (*e.g.*, WNT, BMP, NOTCH) that regulate their fate and spatial organization. Furthermore, differential adhesion properties between cells also contribute to the segregation and patterning observed during development (Gumbiner, 1996). Tissue polarity, in particular, emerges from coordinated cell signaling, cell-cell interactions, and cytoskeletal rearrangements. Signaling pathways, such as planar cell polarity (PCP), regulate the establishment and maintenance of tissue polarity (Jones and Chen, 2007).

In organoids, external stimuli such as growth factors, cytokines, or mechanical cues can be added to modulate pattern formation (Karzbrun et al., 2021) and contribute to the orientation and alignment of cells within the organoids (Silva et al., 2021). Studying tissue polarity in organoids could be useful for deciphering the mechanisms underlying tissue morphogenesis, cell differentiation, and cell polarity establishment. Therefore, manipulating the culture environment, modifying the composition of the culture medium, or applying physical forces can be employed to influence the organization and patterning of cells within organoids. Particularly, modulating signaling pathways or providing specific biochemical or mechanical cues can direct the establishment of tissue polarity in engineered organoids (Silva et al., 2019). Understanding pattern formation in organoids provides insights into the fundamental principles of organ development, including cell fate specification, tissue morphogenesis, and spatial organization. It can also shed light on developmental disorders (Lancaster et al., 2013) and facilitate the engineering of functional tissues or organs for regenerative medicine applications (Lou and Leung, 2018). This research could have implications for modeling diseases related to tissue polarity defects, like polycystic kidney disease (Wilson, 2011).

Organoids also exhibit non-linear dynamics, where small changes in the system can have disproportionately large effects on the overall behavior. For instance, altering culture conditions or genetic features can lead to dramatic changes in organoid development, morphology, and functionality (Velazquez et al., 2021). The responses of organoids to external stimuli can also exhibit non-linear behaviors, such as threshold effects and positive feedback loops (Hannezo and Heisenberg, 2019; Lewis et al., 2021). By comparison, non-linearity is also observed in various biological systems, such as gene regulatory networks, where small changes in gene expression levels can result in significant changes in cellular behavior (Steinacher et al., 2016; Manicka et al., 2023).

Considering organoids as complex systems also offers the opportunity to model complex diseases in a more physiologically relevant context. It has already been shown that they can recapitulate disease-specific phenotypes [*e.g.*, abnormal neural activity in cerebral organoids derived from patients with neurological disorders (Gomes et al., 2020)]. In fact, disease-related emergent behaviors in organoids often result from genetic or environmental factors influencing cell behavior, signaling pathways, or cellular responses. Disease-associated mutations or environmental triggers can disrupt normal cellular processes, leading to aberrant behaviors or disease-specific phenotypes (Maranga et al., 2020). Therefore, the inclusion of disease-relevant genetic mutations, exposure to disease-associated factors, or manipulation of culture conditions can influence the emergence of disease-specific behaviors in organoids. As a result, they offer a means to investigate disease processes that are challenging to study in animal models or traditional cell culture systems, potentially leading to improved diagnostics, targeted therapies, and advancements in regenerative medicine (Sasai, 2013).

In conclusion, organoids exemplify the characteristics of complex systems by displaying emergent behavior, self-organization, and non-linearity. Notably, pattern formation and tissue polarity are among the emergent behaviors observed, arising from intricate cell-cell communication, cell adhesion, and mechanical forces. External stimuli, including growth factors, chemical gradients, or disease-associated factors, can influence these phenomena. Understanding organoid emergent behaviors has implications for elucidating developmental principles, studying disease mechanisms, and advancing regenerative medicine and personalized therapeutics.

## 3 Methods for studying organoids as complex (bio)systems

This contemporary era underlines the necessity to explore novel methodologies for comprehending the intricacies present in both natural and man-made (bio)systems. This imperative aligns with addressing pressing social and economic issues through scientific advancements. Therefore, a seamless fusion of experimental life sciences with computational sciences and other advanced technologies becomes essential for gaining deeper insights into the complexity of biological phenomena (Linshiz et al., 2012). Central to this effort is the identification of underlying patterns governing intricate physiological or pathological processes, in which organoids can be seen as convenient experimental models (Soares et al., 2023; Tenreiro et al., 2023). Achieving this goal entails the production of advanced experimental techniques, together with predictive mathematical models, complemented by machine learning algorithms capable of capturing the molecular complexity within living systems (Figure 2B). An overview of experimental and computational methods used for characterizing complex systems, and their application to organoids is provided bellow.

Firstly, examples of experimental techniques used to study organoids as complex systems, include single-cell RNA sequencing, live imaging, and an assortment of functional assays. Single-cell RNA sequencing (scRNA-seq) and other single-cell omics techniques, in particular, enable the characterization of gene expression profiles and molecular heterogeneity within organoids at a single-cell resolution (Brazovskaja et al., 2019). This allows researchers to identify distinct cell populations, track cellular trajectories during development, and investigate cellular responses to perturbations or environmental cues (Fleck et al., 2022). However, mapping developmental dynamics with organoids is particularly challenging using standard single-cell omics techniques, particularly because spatial and temporal information of the system is lost when processing samples (Wahle et al., 2023).

Indeed, various 3D imaging and tomography methods, such as confocal microscopy, live-cell imaging, light-sheet microscopy, and electron microscopy, allow researchers to visualize the structural organization and dynamics of organoids at cellular and subcellular levels (Nowzari et al., 2021). These high-resolution 3D images of organoids provide insights into cellular behaviors, cell-cell interactions, and the spatial distribution of specific markers or molecules within organoids (D’Imprima et al., 2023). Such capabilities allow researchers to investigate spatial organization, cellular architectures, and complex tissue morphologies. Combining single-cell transcriptomics with spatial imaging has already been tested to explore clonality and lineage dynamics during cerebral organoid development (He et al., 2022). He and coworkers used cellular barcoding, scRNA-seq, and light-sheet microscopy to achieve spatial lineage recordings in cerebral organoids and confirm regional clonality in the developing neuroepithelium. Additional organoid-specific functional assays have also been developed for studying organoids. For example, in brain organoids, electrophysiological measurements, calcium imaging, or multi-electrode array recordings can assess neuronal activity and network properties (Passaro and Stice, 2021; Sharf et al., 2022). Additionally, in intestinal organoids, functional assays can measure barrier function, nutrient absorption, or drug response, providing insights into physiological activities (Zietek et al., 2020).

Moreover, computational modeling approaches, including ordinary differential equations (ODEs), partial differential equations (PDEs), agent-based models, and network modeling, can simulate and predict the behaviors of complex systems like organoids (Gonçalves and García-Aznar, 2023; Pleyer and Fleck, 2023; Wen and Chaolu, 2023). These models integrate known biological mechanisms and parameters to study emergent properties, test hypotheses, and explore the effects of perturbations on organoid development, functionality, and response to external factors (Montes-Olivas et al., 2019). For example, models of intestinal organoids have been developed to investigate the distribution of cell populations and growth patterns in response to signaling dynamics (Buske et al., 2012; Thalheim et al., 2018), to study the biomechanical interactions between cells in crypts (Langlands et al., 2016; Almet et al., 2018), and to evaluate the effect of exogenous substances in the growth pattern of colon cancer organoids (Yan et al., 2018). Additionally, mass transport models have also been used to simulate oxygen and nutrient consumption in cerebral organoids (McMurtrey, 2016; Berger et al., 2018). Finally, reaction-diffusion models have been useful to simulate and predict fate patterning expression in gastruloids (Etoc et al., 2016; Tewary et al., 2017). The generated simulations show signaling expression similar to experimental observations, and accurately predict pattern formation *in vitro* models of gastrulation.

Computational methods for analyzing large-scale omics datasets, such as scRNA-seq data, enable the identification of gene regulatory networks, cell type classification, trajectory analysis, and the detection of emergent patterns and states within biological systems (Stanojevic et al., 2022). Bioinformatics tools also assist in integrating diverse datasets, performing statistical analyses, and generating comprehensive visualizations (Hie et al., 2019; Krassowski et al., 2020; Dries et al., 2021). Presently, machine learning algorithms and artificial intelligence techniques can also be employed to analyze complex and high-dimensional data (Webb, 2018; Bhardwaj et al., 2022). These approaches aid in pattern recognition, clustering, prediction, and classification tasks. In the future, they will help uncover hidden relationships, identify novel biomarkers, and predict organoid behaviors or responses to specific conditions or interventions (Shoji et al., 2023).

Both experimental and computational methods have been widely applied to study organoids as complex systems (Poli et al., 2019). These approaches provide insights into organoid development, functionality, disease modeling, and drug discovery (Wang and Hummon, 2021). For instance, single-cell analysis has revealed cellular heterogeneity, identified key signaling pathways, and uncovered novel cell populations within organoids. Mathematical models and computational simulations have aided in understanding emergent behaviors, optimizing culture conditions, and predicting the response of organoids to different stimuli. Machine learning techniques have been used to analyze complex datasets, classify organoid types, and predict drug responses. Overall, the combination of experimental and computational methods enables a comprehensive characterization and understanding of organoids as complex systems, providing valuable insights into their biology, functionality, and translational applications.

## 4 Genome editing and manipulation of organoid (bio)systems

Genetic engineering techniques, such as CRISPR-Cas9, can be applied to introduce specific genetic modifications or reporter genes into cells (Figure 2C). This technology allows researchers to investigate the roles of specific genes, signaling pathways, or cellular behaviors in organoid development, functionality, or disease modeling (Teriyapirom et al., 2021). This capability opens up a range of possibilities in studying organoid behavior and manipulating emergent properties.

For example, by selectively editing genes associated with specific pathways or signaling cascades, researchers can study the effects of these genetic changes on organoid development, behavior, and function. This helps uncover the underlying genetic mechanisms controlling emergent behavior. Eventually, genome editing can also be employed to promote the generation of specific cell types within organoids and spatially organizing them (Velazquez et al., 2021). This manipulation can recreate tissue-like structures, leading to the development of more sophisticated and accurate models that better mimic the complexity of developing organs (Ho and Morsut, 2021). For example, Cakir and coworkers have engineered human pluripotent stem cells to ectopically express *ETV2* and generate complex vascular-like networks in cerebral organoids (Cakir et al., 2019). Similarly, forced expression of transcription factor *PU.1* also induced the generation of microglia-like cells (Cakir et al., 2022). Overall, the presence of vasculature and microglia enhanced the functional properties of organoids (Cakir et al., 2019; Cakir et al., 2022; Speicher et al., 2022). This cell programming strategy was also used to direct differentiation of human pluripotent stem cells into hepatocytes *in vitro* (Tomaz et al., 2022). The overexpression of three nuclear factors (*HNF1A*, *HNF6*, and *FOXA3*) resulted in the rapid production of hepatocytes with enhanced functional characteristics.

Moreover, by introducing disease-associated mutations into organoids, researchers can study how specific genetic alterations contribute to the development of diseases (Nie and Hashino, 2017). Organoids with such mutations can serve as disease models for testing potential treatments and understanding disease progression (Gomes et al., 2020; Maranga et al., 2020). Organoids can also be derived from individual patients, and genome editing allows for the introduction of specific mutations associated with the patient disease (Li et al., 2020). This personalized approach enables the testing of various drug treatments on patient organoids, potentially leading to more effective and tailored therapies (Shiihara et al., 2021).

Nevertheless, it is important to note that the complexity of emergent behavior in organoids means that controlling it solely through genome editing might not be straightforward, and other factors, such as environmental cues and cellular interactions, also play essential roles. As research in genome editing continues to evolve, further developments into controlling emergent behavior in organoids are expected.

## 5 Future directions and conclusion

As discussed in previous sections, organoids have proven to be powerful tools, replicating the structural and functional complexity of organs *in vitro* (Pașca, 2018). However, future developments are not without obstacles. Reproducibility, scalability, and physiological relevance are key challenges that must be addressed to maximize the impact of organoids in various applications. To overcome these limitations, researchers need to embrace cutting-edge technologies (Gjorevski et al., 2022), such as microfluidics, and advanced imaging techniques. Moreover, involving experts from diverse fields, including biology, engineering, and computational science, can enrich our understanding of organoid development and function.

In this review, I have examined how organoids have the potential to revolutionize our understanding of development, disease, and regenerative medicine. Their ability to recapitulate complex phenomena offers unique opportunities to study human biology in unprecedented ways. From modeling diseases and drug responses, to paving the way for personalized medicine, organoids hold immense promise for the future of healthcare. As we conquer the challenges of reproducibility, scalability, and physiological relevance, the impact of organoids on medical research will undoubtedly flourish. By embracing this technology, we can usher in an era of precise and personalized medicine, ultimately improving the lives of countless individuals worldwide.
